# Comparative mitogenome analyses of twelve non-biting flies and provide insights into the phylogeny of Chironomidae (Diptera: Culicomorpha)

**DOI:** 10.1038/s41598-023-36227-9

**Published:** 2023-06-06

**Authors:** Xiangliang Fang, Xinhua Wang, Bin Mao, Yunli Xiao, Mi Shen, Yue Fu

**Affiliations:** 1 Hubei Key Laboratory of Economic Forest Germplasm Improvement and Resources Comprehensive Utilization, Hubei Collaborative Innovation Center for the Characteristic Resources Exploitation of Dabie Mountains, Hubei Zhongke Research Institute of Industrial Technology, College of Biology and Agricultural Resources, Huanggang Normal University, Huanggang City, 438000 Hubei People’s Republic of China; 2 College of Life Sciences, Nankai University, Tianjin, 300071 China

**Keywords:** Evolution, Systems biology, Zoology

## Abstract

The family Chironomidae is represented by seven subfamilies in China, among which Chironominae and Orthocladiinae are the most diverse. To gain a better understanding of the architecture and evolution of the mitogenomes of Chironomidae, we sequenced mitogenomes of twelve species (including two published species) of the two subfamilies Chironominae and Orthocladiinae, and comparative mitogenomic analyses were performed. Thus, we identified highly conserved genome organization of twelve species with regard to genome content, nucleotide and amino acid composition, codon usage, and gene characteristics. The *K*_*a*_*/K*_*s*_ values of most protein-coding genes were far smaller than 1, indicating that these genes were evolving under purifying selection. Phylogenetic relationships between the family Chironomidae were reconstructed using 23 species representing six subfamilies, based on protein-coding genes and rRNAs using Bayesian Inference and Maximum Likelihood. Our results suggested the following relationship within the Chironomidae: (Podonominae + Tanypodinae) + (Diamesinae + (Prodiamesinae + (Orthocladiinae + Chironominae))). This study contributes to the mitogenomic database of Chironomidae, which will be significant for studing the mitogenome evolution of Chironomidae.

## Introduction

The mitochondrial genome of animals is generally a small (15–20 kb) and comprises 37 genes, i.e., 13 protein-coding genes (PCGs), 22 transfer RNA genes (tRNAs), the large and small ribosomal RNA unit genes (rrnL and rrnS, respectively). There is typically also a single large non-coding region which contains controlling elements for replication and transcription, and various spacer and overlapping regions occur in the mitochondrial genome^1–4^. Unlike the nuclear genome, mitogenomes are typically inherited maternally, they occur at hundreds to thousands of copies per cell^5^, and they exhibit very little recombination in animals^6,7^. Furthermore, the comparison of animal mitochondrial gene arrangements has become a powerful means for inferring long-term evolutionary relationships as rearrangements appear to be unique and generally rare events that are unlikely to arise independently in separate evolutionary lineages^1^.

Chironomids (Diptera: Chironomidae) are important aquatic insects, and they are widely distributed in all biogeographic regions of the world. Further, chironomid larvae are commonly used bioindicators of freshwater ecosystems^8^. The study of the systematic evolution of Chironomidae was initially based on morphology^9,10^, however, modern molecular genetics has further resolved taxonomic diversity and phylogenetics, mainly through genetic markers including mitochondrial sequences^11–16^. In addition, the Chironomidae are a large group of Diptera insects, with more than 6300 known species, however, only few complete Chironomidae mitogenomes have been published so far^17–26^. Previous studies primarily provide descriptions of single mitochondrial genomes or comparative analyses of mitogenomes within genera or among subfamilies. However, there are few mitogenomes and comparative studies of the most diverse subfamilies Orthocladiinae and Chironominae. In addition, some controversies remain with regard to the phylogenetic relationships within the Chironomidae.

In this study, we present novel insights into the phylogenetic relationships between different Chironomidae subfamilies. Furthermore, we performed comparative analyses of mitogenome structure, base composition, and evolutionary rates among twelve species representing the subfamilies Orthocladiinae and Chironominae. Using previously published mitogenomes of the subfamilies Prodiamesinae, Podonominae, Tanypodinae, and Diamesinae, we employed mitochondrial genes isolated from complete mitogenomes to explore the phylogeny of Chironomidae. Further, we evaluate the monophyly of Orthocladiinae and propose to transfer the genus *Propsilocerus* from Orthocladiinae to Prodiamesinae.

## Material and methods

### Sampling and DNA extraction

We included ten previously unpublished Chironomidae mitogenomes and two published completed mitogenomes (i.e., *Chironomus nipponensis*^23^ and *Polypedilum nubifer*^24^). Furthermore, 11 previously published Chironomidae mitochondrial genome sequences were retrieved from NCBI Genbank. In total, 23 Chironomidae species were used as ingroups, including 12 of which were Chironominae, 5 Orthocladiinae, 3 Prodiamesinae, 1 Podonominae, 1 Tanypodinae, and 1 Diamesinae. Collection information and accession numbers of all specimens are shown in Appendix S1. Samples were identified based on morphological descriptions and were preserved and stored in 85% ethanol. Outgroups were selected based on published phylogenetic hypotheses in the Culicomorpha that consider Chironomidae closely related to Ceratopogonidae and Simuliidae^27,28^. All specimens sequenced for this study were deposited in College of Biology and Agricultural Resources, Huanggang Normal University, Hubei Province, China. Genomic DNA was extracted from three to five adult specimens pooled in one centrifuge tube using the TIANamp Micro DNA Kit (TIANGEN Biotech, Beijing, China), according to the manufacturer’s instructions.

### Sequencing and genome assembly

Paired-end sequencing libraries with an insert size of 350 bp were constructed from purified DNA extracts according to a standard protocol for Illumina DNA library construction. Sequencing was carried out using an Illumina NovaSeq 6000 platform (Illumina, San Diego, CA, USA) by a commercial service provider (Origingene, Shanghai, China). A quality assessment of raw FASTQ files of the two libraries was carried using FastQC v0.11.8 (http://www.bioinformatics.babraham.ac.uk/projects/fastqc/). Adapter sequences were removed using Trimmomatic v0.30^29^, and approximately 2 Gb clean data were retained per library after trimming. The original sequences were filtered to obtain high-quality clean sequences which were then assembled using spades v.3.11.1^30^ and Getorganells^31^.

### Data analyses

After assembly, MITOS^32^ and ORF finder (https://www.ncbi.nlm.nih.gov/orffinder/) were used for annotation of PCGs and ribosomal RNA (rRNA) genes. Then, NCBI Blastp and Blastn (*e-value* < 0.001, identity > 90%) were applied to compare PCGs and rRNA genes of mitogenomes of related species reported previously. ARWEN1.2^33^ (http://mbio-serv2.mbioekol.lu.se/ARWEN/) and tRNAscan-SE 2.0^34^ were used to annotate tRNAs.

Mitogenome maps were produced using CG View server 1.0^35^. MEGA X^36^ and Codonw 1.4.4 were used for statistical analyses of base composition, codon usage, and relative synonymous codon usage (RSCU). MISA^37,38^ was used for the detection of simple sequence repeats (SSRs) throughout the genomes. DnaSP 6.12.03^39^ was used for the analysis of non-synonymous substitution rates (K_a_) and synonymous substitution rates (*K*_*s*_). Nucleotide composition bias was calculated according to the AT-skew = (A−T)/(A+T) and GC-skew = (G−C)/(G+C), as previously reported^40^. The AT-Skew and GC-Skew data were normalized for visualization using R (package pheatmap).

### Phylogenetic analyses

Phylogenetic analyses were performed in Bayesian Inference and Maximum Likelihood using all PCGs and rRNA genes in PhyloSuite^41^ with several plug-in programs: the first procedure is to standardize synonymous gene names and identify problematic annotation features, followed by sequence extraction; thereafter, nucleotide and amino acid sequences of genes were individually aligned using Mafft v7.407^42^ with the normal alignment mode and trimmed using Gblocks v0.91^43^ with default settings to remove ambiguously aligned regions. Alignments of 13 PCGs and 2 rRNAs were then concatenated into a supermatrix with the function ‘concatenate sequences’, additionally, this function can record the index of each gene during concatenation and generate a partition file, which can be used in the PartitionFinder software. Then, PartitionFinder2^44^ selected best-fit partitioning schemes and models for the concatenated sequences based on the AICc criterion. Maximum likelihood (ML) analyses using partition mode were performed using IQ-TREE 1.6.8^45^, bootstrapping using ultrafast^46^, and the nodal support values were calculated with 5000 bootstrap replicates. Bayesian inference (BI) analysis was performed using MrBayes 3.2.6^47^ with the partition models. Markov chain Monte Carlo runs of 200,000 generations were conducted, and trees were sampled every 1000 generations with the first 25% of the trees discarded as burn-in. *Anopheles quadrimaculatus* (NCBI accession NC000875), *Wyeomyia confusa* (MK575492), *Simulium variegatum* (NC033348), *Simulium maculatum* (NC040120), *Forcipomyia* sp. (MK000395), and *Culicoides arakawae* (NC009809) were used as outgroups. Tree topology was visualized using iTOL^48^.

## Results

### General structure, organization, and composition of mitogenomes

**Figure 1:**
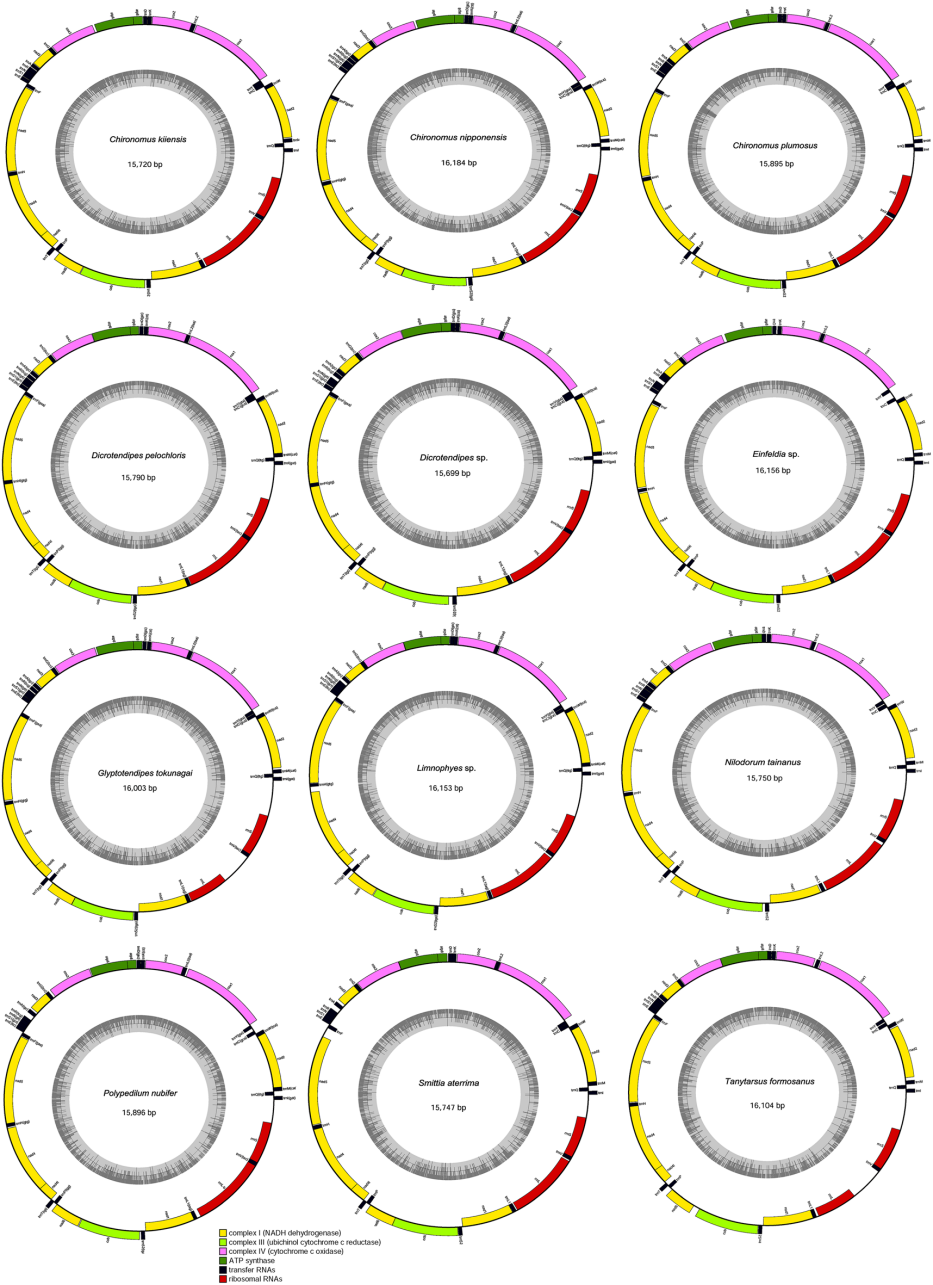
Mitogenome maps of 12 species of Chironomidae.

The gene order and arrangement characteristics of the twelve mitogenomes in the present study (*Chironomus kiiensis*, *Chironomus nipponensis*, *Chironomus plumosus*, *Dicrotendipes pelochloris*, *Dicrotendipes* sp., *Einfeldia* sp., *Glyptotendipes tokunagai*, *Limnophyes* sp., *Nilodorum tainanus*, *Polypedilum nubifer*, *Smittia aterrima*, and *Tanytarsus formosanus*) were similar to those of other chironomids reported previously (e.g.,^20,22,25,26^. The mitogenomes were circular and comprised 13 PCGs, 2 rRNA genes, and 22 tRNA genes (Fig. 1). Mitogenome length ranged from 15,699 bp in *Dicrotendipes* sp. to 16,184 bp in *Chironomus nipponensis* (Table 1). Most of the size variation was due to differences in the control region, and some genomes showed additional noncoding regions within the coding region. Among these genes, four PCGs (ND1, ND4, ND4L, and ND5), eight tRNAs (trnQ, trnC, trnY, trnF, trnH, trnP, trnL, and trnV), and two rRNAs (12S rRNAs and 16S rRNAs) were encoded by the minority strand (N strand), while the other 23 genes were located on the majority strand (J strand) (Appendix S2). ATP8-ATP6 and ND4L-ND4 overlapped by seven nucleotides (ATGATAA and ATGTTAA, respectively) in all twelve Chironomidae species.

**Table 1:** Nucleotide composition of mitochondrial genomes of the 12 Chironomidae species.

Species	Region	Size (bp)	A (%)	T (%)	G (%)	C (%)	A + T (%)	AT-skew	GC-skew
*Chironomus kiiensis*	Whole genome	15,720	39.28	37.6	9.51	13.61	76.88	0.021928	− 0.17744
	tRNAs	1501	41.31	38.11	11.53	9.06	79.42	0.040268	0.119741
	PCGs	11,145	30.17	43.85	12.79	13.18	74.02	− 0.18473	− 0.01485
	1st codon position	3715	31.0902	37.3082	18.9502	12.6514	68.3984	− 0.09134	0.199319
	2nd codon position	3715	20.2692	47.4024	13.2974	19.031	67.6716	− 0.40072	− 0.17735
	3rd codon position	3715	39.1655	46.8371	6.13728	7.86003	86.0027	− 0.0892	− 0.12524
	12S rRNAs	817	41.74	41	11.63	5.63	82.74	0.008876	0.347518
	16S rRNAs	1338	41.55	43.05	10.46	4.93	84.6	− 0.01767	0.359223
	Control region	69	47.95	50.68	1.37	0	98.63	− 0.02778	1
*Chironomus nipponensis*	Whole genome	16,184	39.04	37.06	9.4	14.48	76.1	0.025982	− 0.21262
	tRNAs	1495	41.27	37.99	11.57	9.16	79.26	0.04135	0.116129
	PCGs	11,214	29.04	43.62	13.32	14.01	72.66	− 0.20079	− 0.02512
	1st codon position	3738	30.7384	37.1322	19.1279	13.0016	67.8705	− 0.09464	0.190674
	2nd codon position	3738	20.3104	47.498	13.2727	18.9189	67.8084	− 0.40071	− 0.17539
	3rd codon position	3738	36.0621	46.2547	7.57089	10.1124	82.3167	− 0.12354	− 0.14372
	12S rRNAs	822	41.85	40.88	11.68	5.6	82.73	0.011765	0.352113
	16S rRNAs	1336	42.29	42.37	10.55	4.72	84.66	− 0.00088	0.382353
	Control region	152	39.47	55.92	2.63	1.97	95.39	− 0.17241	0.142857
*Chironomus plumosus*	Whole genome	15,895	38.91	36.64	9.88	14.56	75.55	0.030061	− 0.19146
	tRNAs	1497	41.08	38.34	11.62	8.95	79.42	0.034483	0.12987
	PCGs	11,106	29.05	43.76	13.14	14.06	72.81	− 0.20208	− 0.03377
	1st codon position	3702	31.0103	36.5748	19.1248	13.2901	67.5851	− 0.08277	0.18
	2nd codon position	3702	20.2863	47.2447	13.3712	19.0978	67.5311	− 0.39896	− 0.17637
	3rd codon position	3702	35.8455	47.4608	6.91518	9.7785	83.3063	− 0.13915	− 0.17152
	12S rRNAs	821	42.75	41.17	10.6	5.48	83.92	0.018868	0.318182
	16S rRNAs	1326	42.01	42.61	10.71	4.68	84.62	− 0.00713	0.392157
	Control region	136	36.76	60.29	1.47	1.47	97.05	− 0.24242	0
*Dicrotendipes pelochloris*	Whole genome	15,790	40.23	38.43	8.59	12.74	78.66	0.022945	− 0.19442
	tRNAs	1491	41.05	38.9	11.13	8.92	79.95	0.026846	0.110368
	PCGs	11,214	30.92	45.02	11.88	12.18	75.94	− 0.18577	− 0.0126
	1st codon position	3738	31.4874	37.6672	18.7801	12.0653	69.1546	− 0.08978	0.217693
	2nd codon position	3738	20.3317	47.6458	13.0551	18.9674	67.9775	− 0.40157	− 0.18463
	3rd codon position	3738	40.931	49.7592	3.79882	5.51097	90.6902	− 0.09708	− 0.18391
	12S rRNAs	814	41.89	41.52	11.06	5.53	83.41	0.004418	0.333333
	16S rRNAs	1372	41.55	43.73	9.84	4.88	85.28	− 0.02564	0.336634
	Control region	682	47.69	50.38	1.54	0.38	98.07	− 0.02745	0.6
*Dicrotendipes* sp.	Whole genome	15,699	39.76	36.25	9.36	14.63	76.01	0.046174	− 0.21933
	tRNAs	1498	40.85	38.99	11.62	8.54	79.84	0.023411	0.152318
	PCGs	11,217	29.5	43.05	13.25	14.2	72.55	− 0.18678	− 0.03475
	1st codon position	3739	30.8639	36.8815	19.096	13.1586	67.7454	− 0.08926	0.18408
	2nd codon position	3739	20.4065	47.7668	13.1586	18.6681	68.1733	− 0.4011	− 0.17311
	3rd codon position	3739	37.2292	44.5039	7.48863	10.7783	81.7331	− 0.08871	− 0.18009
	12S rRNAs	820	40.12	42.2	12.07	5.61	82.32	− 0.02519	0.365517
	16S rRNAs	1361	40.93	43.86	10.73	4.48	84.79	− 0.03466	0.410628
	Control region	413	45.52	52.06	0.48	1.94	97.58	− 0.067	− 0.6
*Einfeldia* sp.	Whole genome	16,156	40.13	39.32	8.37	12.18	79.45	0.010284	− 0.1853
	tRNAs	1496	41.78	38.24	11.1	8.89	80.02	0.044277	0.110368
	PCGs	11,106	31.04	45.5	11.97	11.49	76.54	− 0.18894	0.020345
	1st codon position	3702	31.1453	38.6008	18.9357	11.3182	69.7461	− 0.10732	0.251786
	2nd codon position	3702	20.5024	47.4068	13.1551	18.9357	67.9092	− 0.39594	− 0.18013
	3rd codon position	3702	41.4753	50.4999	3.80978	4.21508	91.9751	− 0.09785	− 0.05051
	12S rRNAs	813	41.94	41.82	10.7	5.54	83.76	0.001468	0.318182
	16S rRNAs	1351	43.6	41.3	10.51	4.59	84.9	0.027027	0.392157
	Control region	231	41.13	58.01	0.87	0	99.14	− 0.17031	1
*Glyptotendipes tokunagai*	Whole genome	16,003	40.15	37.09	8.52	14.24	77.24	0.039644	-0.25151
	tRNAs	1487	41.56	38.4	11.37	8.68	79.96	0.039529	0.134228
	PCGs	11,220	30.07	43.98	12.37	13.57	74.05	− 0.18777	− 0.04638
	1st codon position	3740	31.5775	37.0856	18.5294	12.8075	68.6631	− 0.08064	0.182594
	2nd codon position	3740	20.3209	47.8342	13.1818	18.6631	68.1551	− 0.40345	− 0.17212
	3rd codon position	3740	38.3258	47.0179	5.40251	9.25381	85.3437	− 0.10157	− 0.26277
	12S rRNAs	821	42.27	41.66	10.72	5.36	83.93	0.007257	0.333333
	16S rRNAs	1369	41.86	43.61	9.79	4.75	85.47	− 0.02051	0.346734
	Control region	290	47.31	50.38	0.77	1.54	97.69	− 0.0315	− 0.33333
*Limnophyes* sp.	Whole genome	16,153	40	37.35	9.66	12.99	77.35	0.034334	− 0.14707
	tRNAs	1482	40.82	38.39	12.21	8.57	79.21	0.030664	0.175325
	PCGs	11,196	30.59	43.49	13.04	12.88	74.08	− 0.1741	0.006203
	1st codon position	3732	31.0289	37.5402	19.239	12.1919	68.5691	− 0.09539	0.224211
	2nd codon position	3732	20.686	46.0879	13.8264	19.3998	66.7738	− 0.38017	− 0.16774
	3rd codon position	3732	40.0589	46.8382	6.05573	7.04716	86.8971	− 0.07835	− 0.07566
	12S rRNAs	837	40.02	42.77	10.87	6.33	82.79	− 0.03319	0.263889
	16S rRNAs	1349	39.88	43.74	10.67	5.71	83.62	− 0.0461	0.303167
	Control region	248	45.56	50.81	1.61	2.02	96.37	− 0.05439	− 0.11111
*Nilodorum tainanus*	Whole genome	15,750	40.11	38.64	8.67	12.57	78.75	0.018704	− 0.18326
	tRNAs	1498	41.46	38.85	11.28	8.41	80.31	0.032419	0.145763
	PCGs	11,106	30.68	45.35	12.16	11.8	76.03	− 0.19304	0.015408
	1st codon position	3702	31.2264	37.8444	18.8277	12.1016	69.0708	− 0.09624	0.217467
	2nd codon position	3702	20.4214	47.4068	13.2091	18.9627	67.8282	− 0.39761	− 0.17884
	3rd codon position	3702	40.3945	50.8241	4.45825	4.32316	91.2186	− 0.11407	0.015385
	12S rRNAs	822	43.43	40.88	10.34	5.35	84.31	0.030303	0.317829
	16S rRNAs	1299	43.19	41.57	10.39	4.85	84.76	0.019074	0.363636
	Control region	291	48.45	48.45	1.37	1.72	96.9	0	− 0.11111
*Polypedilum nubifer*	Whole genome	15,896	40.68	36.32	8.54	14.46	77	0.056699	− 0.25711
	tRNAs	1488	41.26	39.85	10.95	7.93	81.11	0.017399	0.160142
	PCGs	11,217	30.08	43.51	12.53	13.87	73.59	− 0.18256	− 0.05064
	1st codon position	3739	31.9604	36.9885	18.2134	12.8377	68.9489	− 0.07334	0.173127
	2nd codon position	3739	20.46	47.8738	13.0249	18.6413	68.3338	− 0.40094	− 0.17736
	3rd codon position	3739	37.8176	45.6807	6.36534	10.1364	83.4983	− 0.09388	− 0.22853
	12S rRNAs	810	42.1	42.22	10.25	5.43	84.32	− 0.00146	0.307087
	16S rRNAs	1430	41.26	44.41	9.79	4.55	85.67	− 0.03673	0.365854
	Control region	166	48.8	48.8	1.81	0.60	97.60	0	0.5
*Smittia aterrima*	Whole genome	15,747	39.32	38.24	9.32	13.12	77.56	0.013918	− 0.16954
	tRNAs	1480	42.5	38.38	10.54	8.58	80.88	0.050961	0.102473
	PCGs	11,169	30.57	44.08	12.52	12.84	74.65	− 0.181	− 0.01271
	1st codon position	3723	31.7754	37.7652	18.7483	11.711	69.5407	− 0.08655	0.231041
	2nd codon position	3723	20.2793	46.7634	13.9672	18.9901	67.0427	− 0.39479	− 0.1524
	3rd codon position	3723	39.6454	47.7035	4.83481	7.81628	87.3489	− 0.09197	− 0.23567
	12S rRNAs	785	41.4	40.89	10.7	7.01	82.29	0.006192	0.208633
	16S rRNAs	1318	40.74	43.02	10.62	5.61	83.76	− 0.02717	0.308411
	Control region	374	43.05	53.21	1.07	2.67	96.26	− 0.10556	− 0.42857
*Tanytarsus formosanus*	Whole genome	16,104	41.11	37.53	8.04	13.33	78.64	0.045483	− 0.24767
	tRNAs	1496	41.51	39.1	11.1	8.29	80.61	0.029851	0.144828
	PCGs	11,115	30.51	44.99	12.07	12.42	75.5	− 0.19185	− 0.01432
	1st codon position	3705	31.9838	39.0283	17.7058	11.2821	71.0121	− 0.09962	0.221601
	2nd codon position	3705	20.5128	47.8003	13.1984	18.4885	68.3131	− 0.39921	− 0.16695
	3rd codon position	3705	39.0283	48.1511	5.31714	7.50337	87.1795	− 0.10437	− 0.17053
	12S rRNAs	832	41.35	43.51	9.98	5.17	84.86	− 0.0255	0.31746
	16S rRNAs	1374	42.94	42.5	4.37	10.19	85.44	0.005111	− 0.4
	Control region	588	48.13	47.79	1.19	2.89	95.92	0.003546	− 0.41667

**Figure 2:**
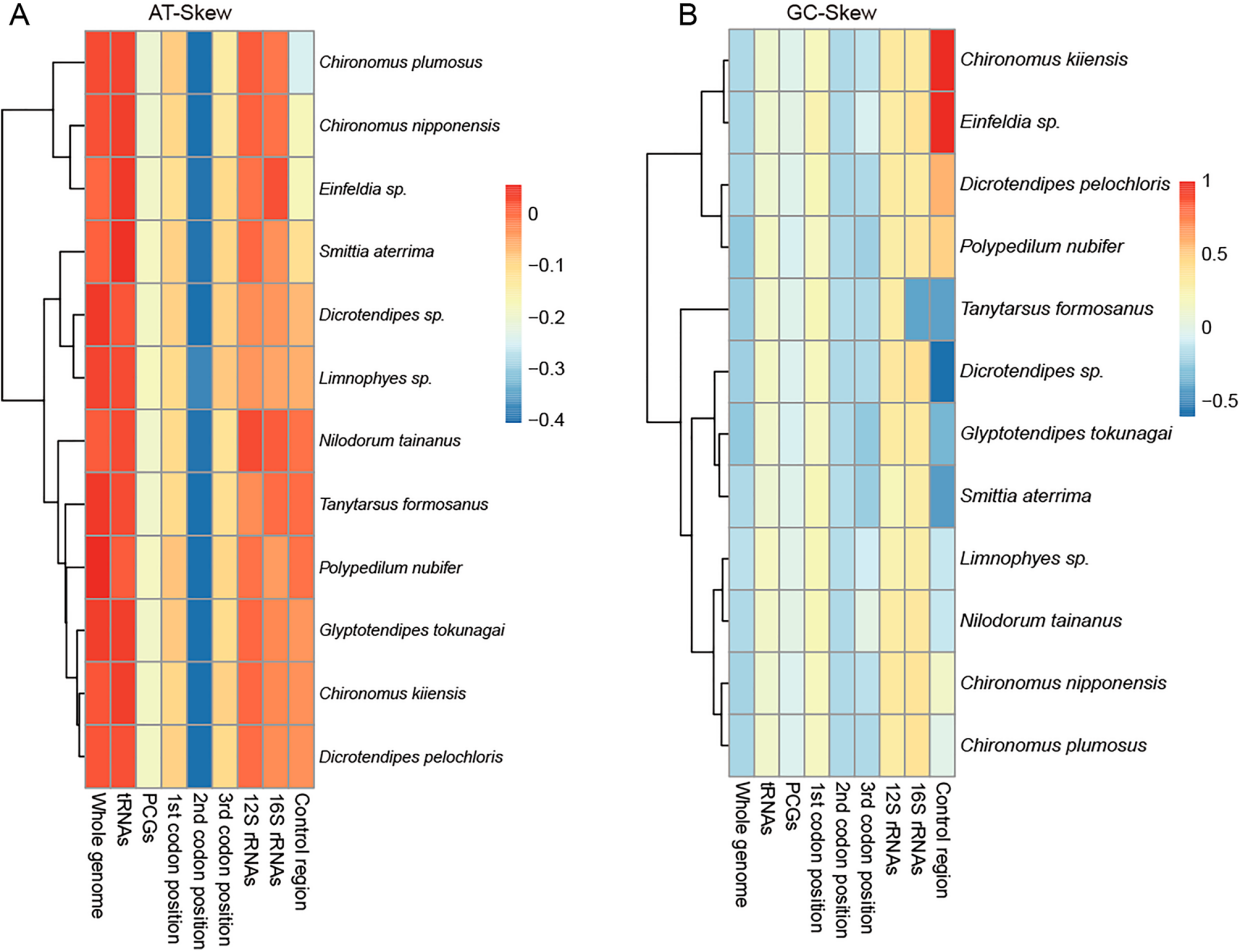
(**A**) *AT-skew* values of various datasets of twelve chironomid mitogenomes. Hierarchical clustering of chironomid species (y-axis) based on AT-skew. (**B**) *GC-skew* values of various datasets of twelve chironomid mitogenomes. Hierarchical clustering of chironomid species (y-axis) based on GC-skew. The legend showed the normalized score of *AT-Skew* and *GC-Skew* values.

All mitogenomes examined here showed base composition biases as are typically observed in insect mitogenomes^49^. The AT content and skew statistics are shown in Table 1, indicating a pronounced A + T bias (75.55%–79.45%). The control region showed the highest A + T content, varying from 95.39% (*Chironomus nipponensis*) to 99.14% (*Einfeldia* sp.), whereas the first and the second codon positions of PCGs had the lowest A + T content, varying from 66.77 to 71.01%. With regard to complete mitogenomes, the *AT-skew* values were positive, varying from 0.010 (*Einfeldia* sp.) to 0.057 (*Polypedilum nubifer*), whereas *GC-skew* values were negative in all species, varying from − 0.257 (*Polypedilum nubifer*) to − 0.147 (*Limnophyes* sp.) (Fig. 2).

**Figure 3:**
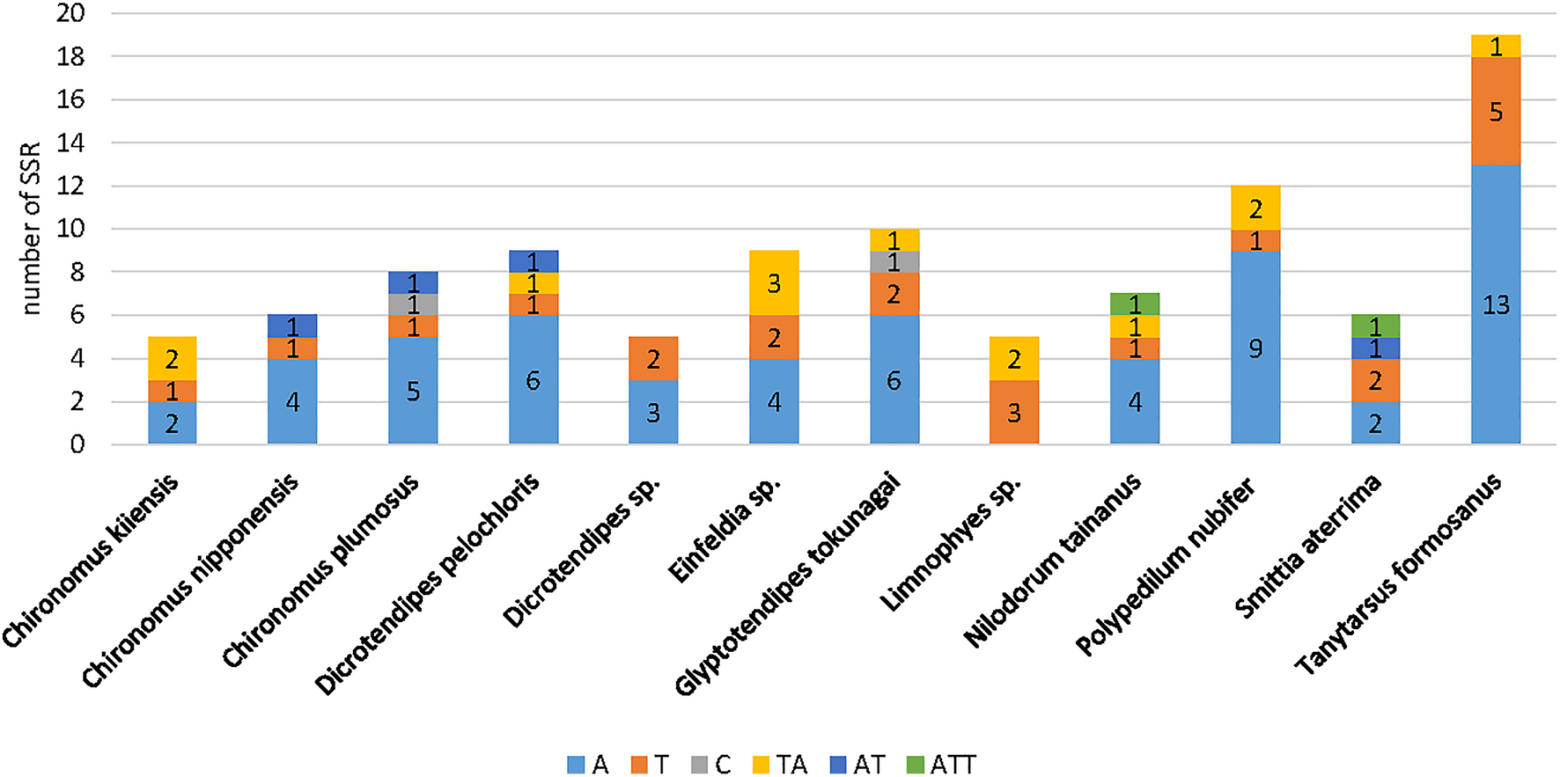
Simple sequence repeats (SSR) in the mitochondrial genomes of 12 species.

Analysis using MISA^37^ showed the presence of 5–19 unequal SSRs in each of the 12 mitogenomes (Fig. 3), and the SSRs were mainly dominated by single nucleotide repeats; the number of dinucleotide repeats of each species was generally 1 or 2 (except for *Dicrotendipes* sp., *Einfeldia* sp.). Trinucleotide repeats occurred only in *Nilodorum tainanus* and *Smittia aterrima*. The single nucleotide was repetitive for A/T, and the dinucleotide repeated only AT/TA types. Trinucleotide repeats showed only ATT repeated, and no CG/GC types were detected. All SSRs contained high ratios of A or T bases.

### Protein-coding genes

The mitogenomes comprised 13 classical PCGs, of which 7 were NADH dehydrogenase subunit genes (*nad*), 3 were cytochrome c oxidase subunit genes (*cox*), 2 were ATP synthase subunit genes (*atp*), and 1 was a cytochrome b gene (*cytb*). The total length of PCGs ranged from 11,106 to 11,220 bp, and the A + T content in this region ranged from 72.55 to 76.54%. The A + T content of the third codon position (82.32–91.98%) was higher compared to that of the first (67.58–71.01%) and second codons (66.77–68.33%) (Table 1).

**Figure 4:**
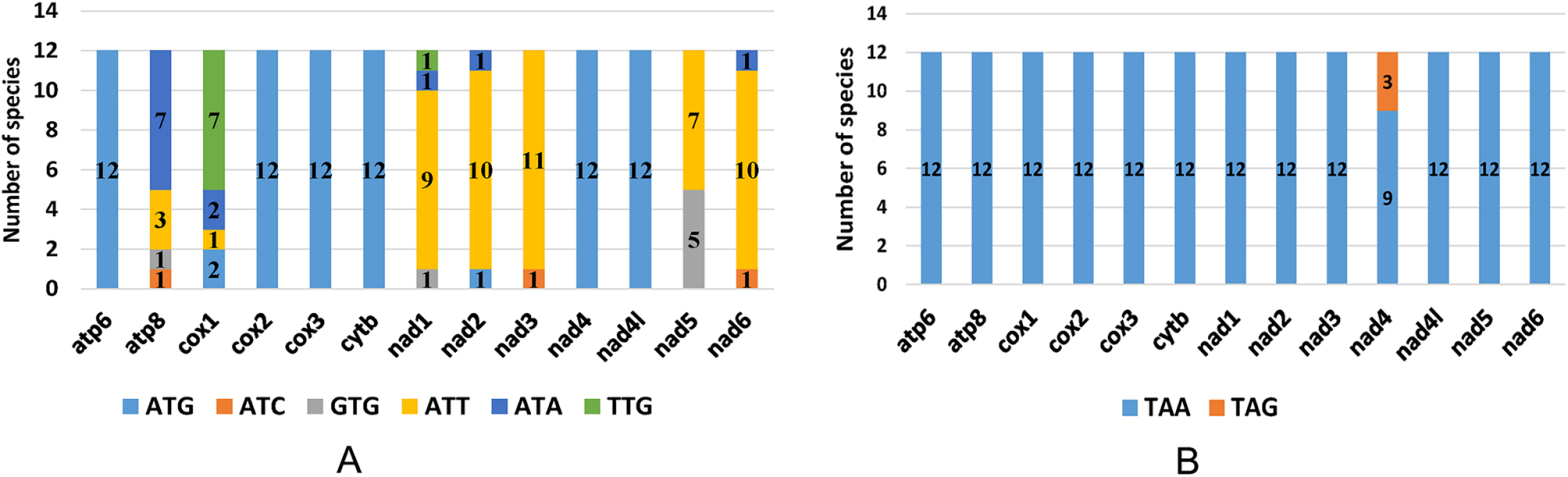
Comparison of start codons (**A**) and stop codons (**B**) of PCGs of twelve species.

Six types of start codons (ATG, ATC, GTG, ATT, ATA, and TTG) were observed (Fig. 4). Among species, the start codons of the same PCG differed; the *atp8* gene started with ATA, ATT, GTG, or ATC in different species; the *coxI* gene started with TTG, ATA, ATT, or ATG; the *nad1* gene started with TTG, ATA, ATT, or GTG; the *nad2* gene started with ATA, ATT, or ATG; the *nad3* gene started with ATT or ATC; the *nad5* gene started with ATT or GTG; the *nad6* gene started with ATA, ATT, or ATC; the *atp6*, *coxII*, *coxIII*, *cytb*, *nad4*, and *nad4L* genes started with ATG. The presence of alternative start codons in mitochondrial genomes can produce implications for gene expression, translation efficiency, and translational accuracy. For example, a TTG start codon variant was identified for the *coxI* gene, rather than the conventional ATN start codon. This variation may led to a decrease in translation efficiency, thus causing incomplete protein expression.

Additionally, there were two types of stop codon: TAA and TAG. The *nad4* gene terminated with TAA or TAG, other genes terminated with TAA (Appendix S2). The length of each PCG was consistent among species (Fig. S1), of which the *nad5* gene was the longest (1600–1800 bp), followed by the *coxI* gene (1500–1600 bp), and the *ATP8* gene was the shortest (< 200 bp).

**Figure 5:**
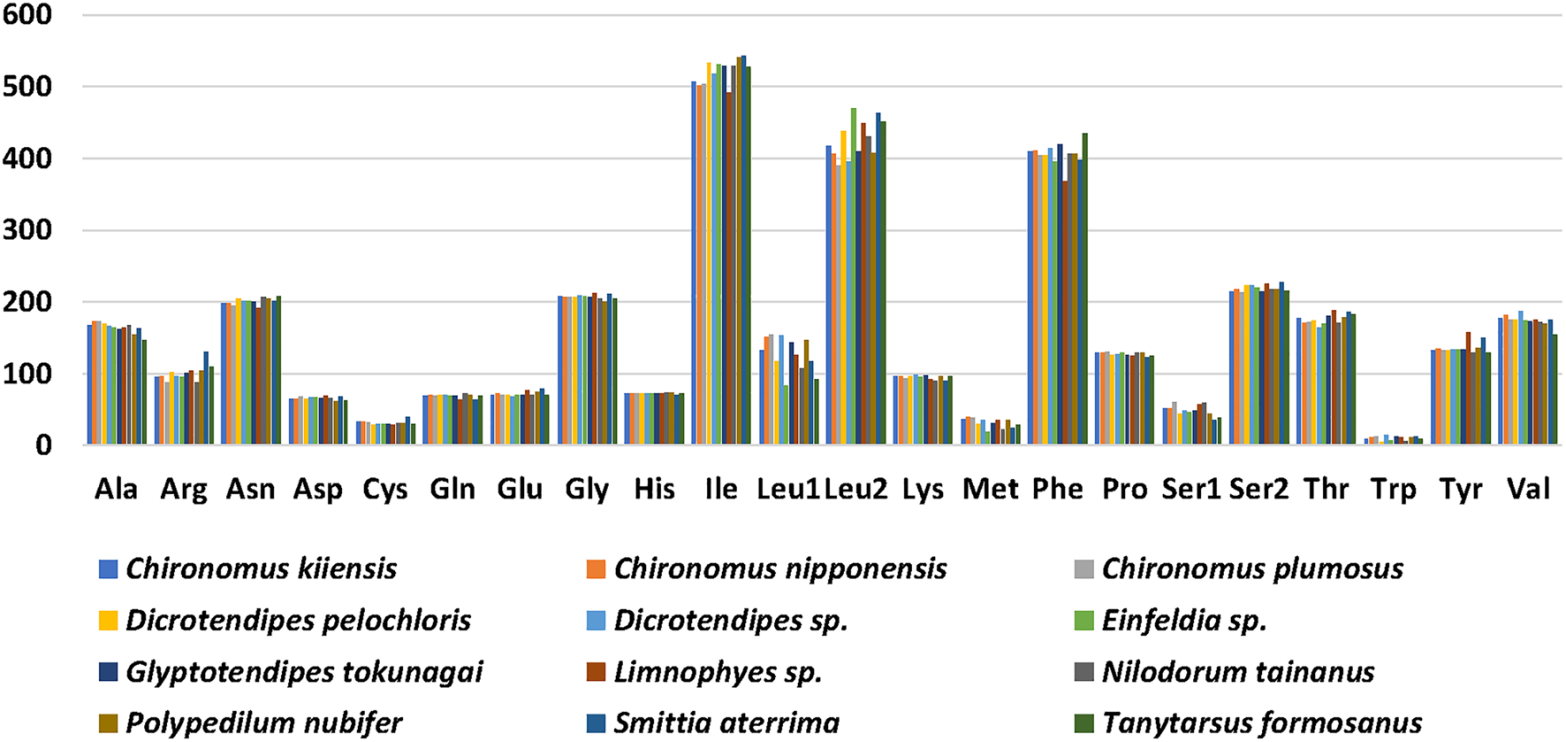
Patterns of codon usage in the 12 mitogenomes. The codon families are shown on the X-axis, and the total codons are shown on the Y-axis.

**Figure 6:**
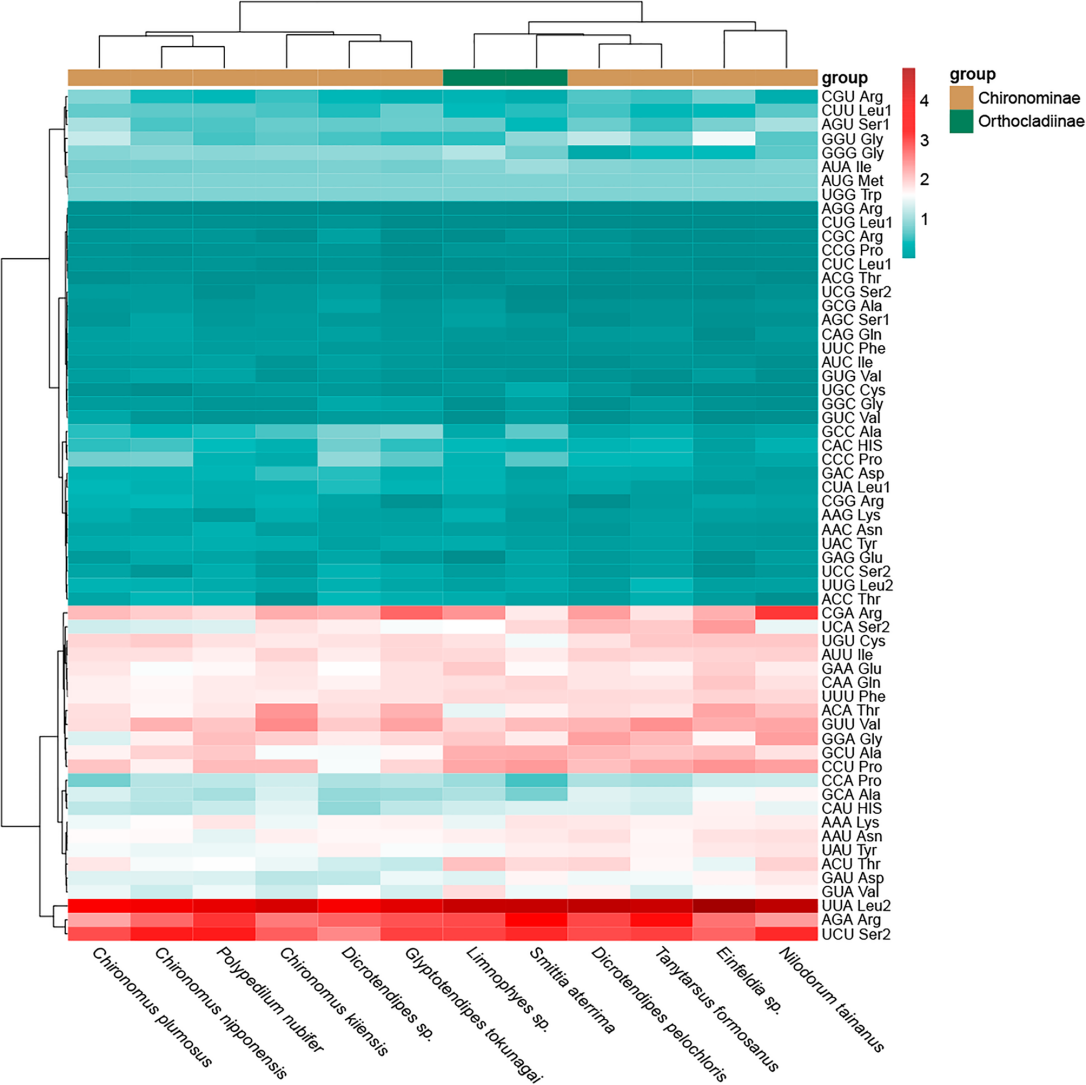
Relative synonymous codon usage (RSCU) for the mitochondrial genomes of twelve species. Codon families and RSCU values are shown on the Y-axis. Hierarchical clustering of chironomid species (x-axis) based on the RSCU are shown on the X-axis.

The total number of codons ranged from 3702 to 3740 in the 13 protein-coding genes excluding the stop codons (Table 1). Codons encoding Ile, Leu2, Phe, and Ser2 (492–543, 390–469, 368–435, and 213–228, respectively) were more abundant compared to codons encoding other amino acids. The codons encoding Trp, Cys, and Met were fewer (5–15, 29–40, and 19–40, respectively) (Fig. 5). The 12 mitogenomes shared the same codon families and had similar characteristics of RSCU. For each amino acid, the most prevalent usage codons were NNA and NNU (Fig. 6), which was consistent with the higher A + T content of the third codon of PCGs.

**Figure 7:**
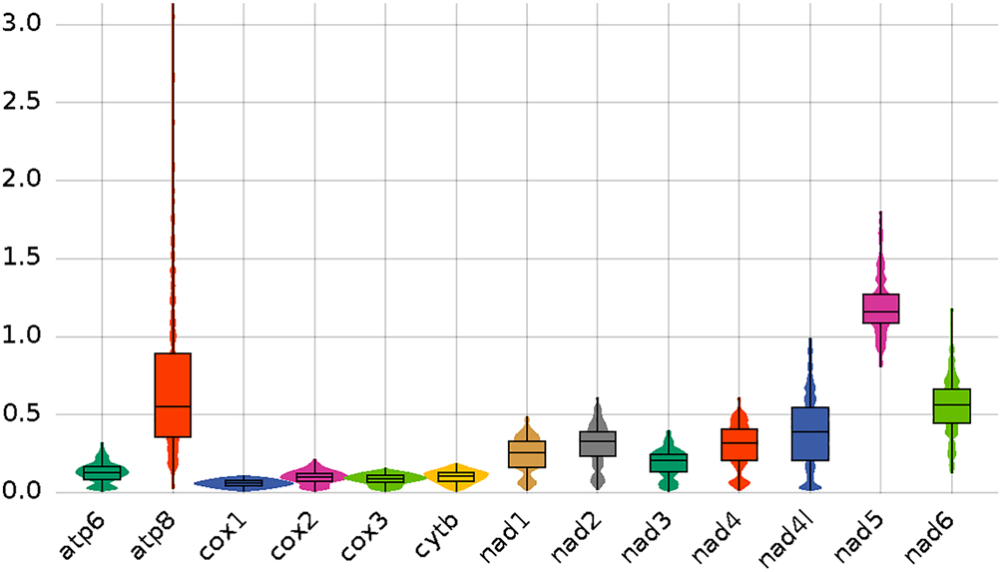
Evolution rate (K_a_/K_s_) of each PCG. The names of PCGs are shown on the X-axis, and *K*_*a*_*/K*_*s*_ values are shown on the Y-axis.

The ratio of K_a_/K_s_ (ω) was used to investigate the evolutionary rates of mitochondrial PCGs (Fig. 7). The results showed that the ω values of the 12 PCGs (except *nad5*) were all below 1.0, indicating a strong repair mechanism against deleterious mutations in most PCGs. However, the K_a_/K_s_ ratio differed significantly among individual genes, implying that the region comprised varying functional constraints. The *K*_*a*_*/K*_*s*_ value of *nad5* was the highest, implying the least purifying selective pressure. *coxI* exhibited the lowest *K*_*a*_*/K*_*s*_ value, indicating the strongest purifying selective pressure.

### tRNAs, rRNAs, and control regions

Twenty-two typical tRNAs were found in twelve Chironomidae species. The number of base pairs in the 22 tRNAs ranged from 64 to 74 bp (trnK) (Appendix S2). The AT content of tRNA genes was A + T-biased, ranging from 79.21% (*Limnophyes* sp.) to 81.11% (*Polypedilum nubifer*). All tRNAs exhibited positive AT-skew and positive GC-skew (Table 1, Fig. 2).

The two genes encoding the ribosomal subunits were located between *trnV* and the control region and between *trnL1* and *trnV*, respectively. The length of the *rrnL* genes ranged from 1299 to 1430 bp, and that of the *rrnS* genes ranged from 785 to 837 bp (Appendix S2). Both 12S rRNA genes and 16S rRNA genes were on the N-strand. The A + T content of 12S rRNA genes ranged from 82.29% (*Smittia aterrima*) to 84.32% (*Polypedilum nubifer*), and that of the 16S rRNA genes ranged from 83.76% (*Smittia aterrima*) to 85.67% (*Polypedilum nubifer*). The 12S rRNA genes exhibited negative a AT-skew in *Dicrotendipes* sp., *Limnophyes* sp., *Polypedilum nubifer*, and *Tanytarsus formosanus* and a positive AT-skew in the other species. The 16S rRNA genes showed a positive AT-skew in *Einfeldia* sp., *Nilodorum tainanus*, and *Tanytarsus formosanus* and a negative AT-skew in the other species. The 12S rRNA genes exhibited a positive GC-skew in all 12 mitogenomes. The 16S rRNA genes showed a negative GC-skew in *Tanytarsus formosanus* and a positive GC-skew in the other mitogenomes (Table 1, Fig. 2).

The control region is rich in A and T bases. It contains the initiation and regulatory elements of mitochondrial DNA transcription and replication and is the largest non-coding region in the mitochondrial genome^4^. The Chironomidae family is a highly diverse group of aquatic insects that possess complex control regions in their mitochondrial DNA. These control regions comprise of various regulatory elements, including conserved sequence blocks, promoter regions, stem-loop structures, polyadenylation sites, and other essential regulatory components. This complex arrangement of regulatory elements offers novel insights into the efficient regulation of mitochondrial function, including transcription and replication, which are necessary for sustaining mitochondrial physiology in Chironomidae and other organisms. In the present study, the length of the control region varied markedly between species, ranging from 69 bp (*Chironomus kiiensis*) to 682 bp (*Dicrotendipes pelochloris*), and it was located between *rrnS* and *trnI*. Its size can impact replication efficiency, and in some cases, smaller control regions may enhance replication efficiency by reducing the time required for the replication machinery to unwind the DNA and initiate replication. Analyses of the control region showed that the proportions of A, T, G, and C were 36.76–48.80%, 47.79–60.29%, 0.48–2.63%, and 0–2.67%, respectively. The control region showed a positive AT-skew in *Nilodorum tainanus*, *Polypedilum nubifer* and *Tanytarsus formosanus* and a negative AT-skew in the other species. The control region exhibited a negative GC-skew in *Dicrotendipes sp.*, *Glyptotendipes tokunagai*, *Limnophyes* sp., *Nilodorum tainanus*, *Smittia aterrima,* and *Tanytarsus formosanus* and a positive GC-skew in the other species (Table 1, Fig. 2).

### Phylogenetic analyses

**Figure 8:**
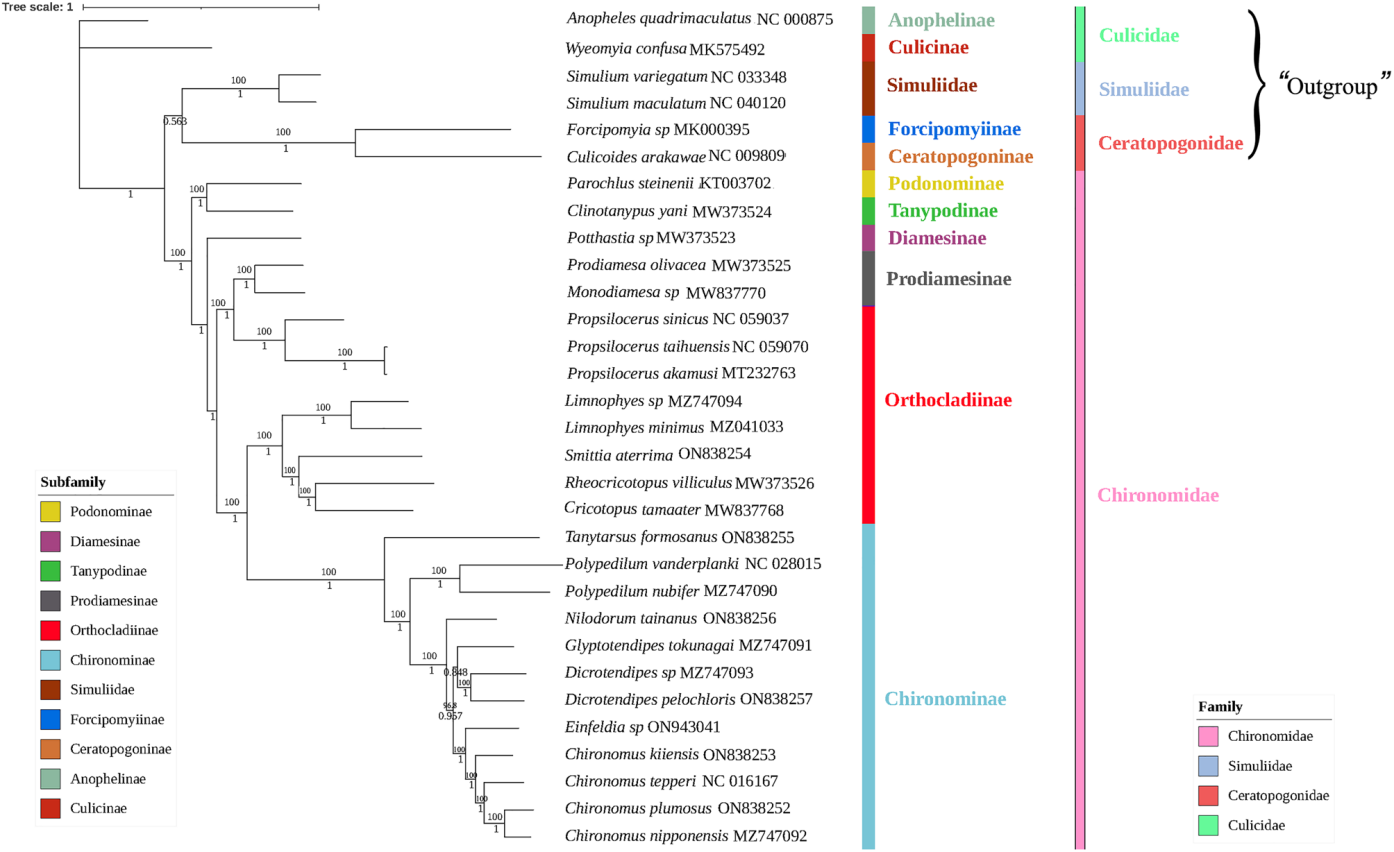
Phylogenetic analysis of Chironomidae and related species based on the data set of PCGs + rRNAs. Numbers on branches represent bootstrap values (> 70%) and whereas those under the branch represent posterior probabilities (> 90%). *Anopheles quadrimaculatus* NC000875, *Wyeomyia confusa* MK575492, *Simulium variegatum* NC033348, *Simulium maculatum* NC040120, *Forcipomyia* sp. MK000395, and *Culicoides arakawae* NC009809 were used as outgroups.

Mitogenomes contain multiple types of valid information appropriate for phylogenetic and evolutionary analyses, such as the amino acid sequence, the secondary structure of RNA, and the arrangement order of genes^50–52^. In the present study, phylogenetic analyses based on the data set of PCGs and rRNAs using ML and BI methods showed similar phylogenetic relationships of six subfamilies within the Chironomidae in terms of topology. Phylogenetics trees (Fig. 8) showed distinct subfamilies and confirmed monophyly, apart from Orthocladiinae and Prodiamesinae. This result strongly supports that the monophyletic *Propsilocerus* of Orthocladiinae is a sister taxon to *Monodiamesa* + *Prodiamesa* of Prodiamesinae, which is consistent with previous results^13,20^. According to Cranston et al. ^13^, *Propsilocerus* probably belongs to the Prodiamesinae rather than to the Orthocladiinae; however, Lin et al.^20^ considered Prodiamesinae a subgroup of Orthocladiinae. Based on our mitogenome phylogenies, it would be appropriate to transfer *Propsilocerus* as a subgroup of Prodiamesinae to make Orthocladiinae monophyletic. In addition, we compared the morphological characters between *Propsilocerus* and species in Prodiamesinae based on previous studies^53,54^ and identified a number of synapomorphies: medium to large species, color generally brown to black, abdomen typically with setae in paler spots; acrostichals typically absent, when present long and starting near scutal projection; R_4+5_ ending distal to end of M_3+4_; hind tibia with outer spur longer than 1/2 length of inner spur; inferior volsella typically conspicuously long and broad, arising from inner margin near base and extending posteriorly approximately to the level of the gonostylus. Combining mitogenomes analysis and morphological evidence, *Propsilocerus* should be transferred from the Orthocladiinae to Prodiamesinae, as proposed by Cranston et al.^13^.

At the base of Chironomide, the subfamilies Podonominae and Tanypodinae are ancestral taxa and were supported (BS = 100, PP = 1) as sister groups to the remaining taxa. Diamesinae (BS = 100, PP = 1) was a sister clade to Prodiamesinae + (Orthocladiinae + Chironominae). Prodiamesinae (BS = 100, PP = 1) was a sister group to Orthocladiinae + Chironominae. In the present study, the mitogenomic data used to explore the phylogenetic relationships of six subfamilies within the Chironomidae generally showed consistent trends with traditional morphology-based systematics^10^. Therefore, we confirm that mitogenomic data are critical for phylogenetic reconstructions at subfamily level in the Chironomidae.

## Conclusions

In the PCGs of 12 mitogenomes, the A + T content of the third codon position was markedly higher than those of the first and second positions, as in the published mitogenomes of other Chironomidae. For the whole mitogenomes, the *AT-skew* values were positive, while the *GC-skew* values were negative in all species, which is in line with the mitochondrial genomes of most insects contained more A than T, while G content was lower than that of C. SSRs mainly comprised mono-, di-, and trinucleotide repeats, with a high proportion of A or T bases. In terms of codon usage in this study, the relative frequency of synonymous codon usage showed that codons ending in A/U were more frequent than those ending in G/C, which was consistent with the high AT characteristics of insect mitochondrial genomes.

In the mitochondrial evolution rate analysis of the current study, the K_a_/K_s_ ratio of all genes (except *nad5*) was < 1, indicating mainly the purification selection of PCGs. The *nad5* was the largest among the 13 PCGs, but it is rarely used in the phylogeny of Chironomidae, likely owing to its rapid rate of evolution. The *coxI* gene was the second-longest, and its *K*_*a*_*/K*_*s*_ value was the lowest; therefore, it provides the strongest and the most conserved effective phylogenetic and evolutionary signals among all genes, and it is frequently used as a key barcode marker to identify species and determine inter-species differences. In general, different *K*_*a*_*/K*_*s*_ values reflected that genes were subjected to different degrees of purification selection.

According to the phylogenetic results, the basic branches of Chironomidae included the subfamilies Podonominae and Tanypodinae. When *Propsilocerus* is transferred to the Prodiamesinae subfamily, all six subfamilies are monophyletic. The phylogenetic relationship of the six subfamilies is thus (Podonominae + Tanypodinae) + (Diamesinae + (Prodiamesinae + (Orthocladiinae + Chironominae))). Obtaining more data on the mitochondrial genome of Chironomidae will provide important support for research on the phylogenetic relationship of Chironomidae.

## Supplementary Information

Supplementary Information 1.

Supplementary Information 2.

Supplementary Figure S1.

Supplementary Figure Legend.

## Data Availability

The following information was supplied regarding data availability: The newly sequenced ten mitochondrial genomes are available at NCBI SRA (BioProject ID: PRJNA899133, PRJNA899132, PRJNA899129, PRJNA899131, PRJNA899130, PRJNA899128, PRJNA898980, PRJNA898895, PRJNA898097, PRJNA752912), and the assembled sequences are available at GenBank (ON838252, ON838253, ON838254, ON838255, ON838256, ON838257, ON943041, MZ747094, MZ747093, MZ747091).
