# Supplementary figures and images for: Comparative mitogenome analyses of twelve non-biting flies and provide insights into the phylogeny of Chironomidae (Diptera: Culicomorpha)
Source: Sci Rep. 2023 Jun 6;13:9200. doi: 10.1038/s41598-023-36227-9 (PMC10244353; doi:10.1038/s41598-023-36227-9)

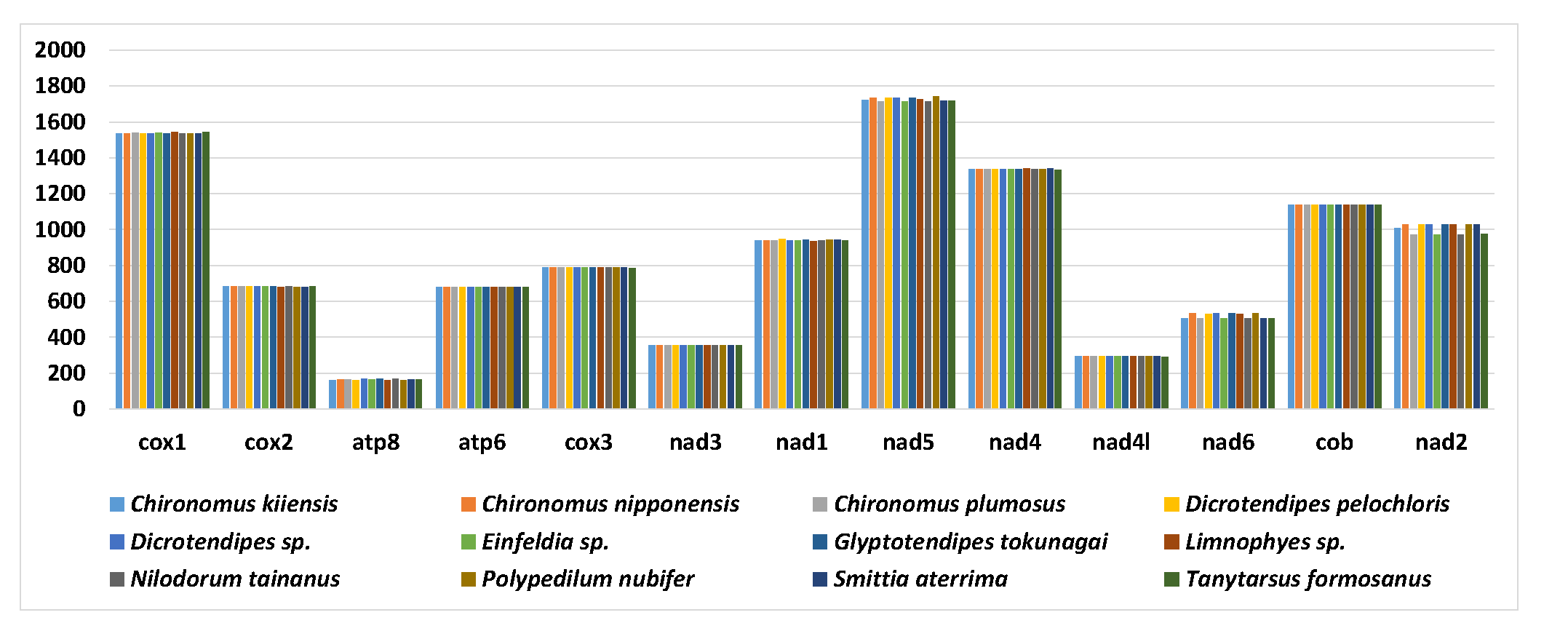

Supplement: Supplementary file 3 — Supplementary Figure S1. [file 41598_2023_36227_MOESM3_ESM.tif]
