# Supplementary material for: Comparative mitogenome analyses of twelve non-biting flies and provide insights into the phylogeny of Chironomidae (Diptera: Culicomorpha)
Source: Sci Rep. 2023 Jun 6;13:9200. doi: 10.1038/s41598-023-36227-9 (PMC10244353; doi:10.1038/s41598-023-36227-9)
Supplement: Supplementary file 4 — Supplementary Figure Legend. [file 41598_2023_36227_MOESM4_ESM.docx]

Figure S1. Length of 13 protein coding genes of the twelve mitogenomes
